# Evolutions of partner-ruled surfaces with simultaneous inextensibility conditions

**DOI:** 10.1371/journal.pone.0336149

**Published:** 2025-12-01

**Authors:** Kemal Eren, Soley Ersoy, Mohammad Nazrul Islam Khan

**Affiliations:** 1 Picode Software, Education Training Consultancy Research and Development and Trade Ltd. Co., Sakarya, Türkiye; 2 Department of Mathematics, Faculty of Sciences, Sakarya University, Sakarya, Türkiye; 3 Department of Computer Engineering, College of Computer, Qassim University, Buraydah, Saudi Arabia; National University of Sciences and Technology, PAKISTAN

## Abstract

Ruled surfaces are a class of surfaces generated by a moving straight line and are used in various design and modeling applications, including kinematics, robotics, and computer-aided geometric design. On the other hand, the inextensibility condition, which preserves the intrinsic geometry of a surface during evolution, is significant for motions of surfaces where stretching is not allowed. Nevertheless, comprehensive characterizations of the evolutions of partner-ruled surfaces generated by the canonical vector pair of a space curve and the preservation of their intrinsic properties under evolutions remain open for investigation. This study aims to provide systematic research on the inextensible evolutions of partner-ruled surfaces simultaneously generated by a pair from the set of Darboux and Frenet vectors of a unit-speed space curve. For this purpose, the intrinsic and extrinsic invariants of the partner-ruled surfaces are derived, and their inextensibility conditions are determined. The structural classifications of the parameter curves of these types of surfaces are explored. The main contributions are the necessary and sufficient conditions on the curvature and torsion of the generator curve, which state the simultaneous inextensibility of these partner surfaces. Additionally, characterizations for the simultaneous developability and minimality of these partner surfaces are provided, based on Gauss and mean curvatures calculated using partial differential equations. This work provides a classification for this family of surfaces, supported by clear visual examples.

## Introduction

In the 18th century, the research on the properties of curves and surfaces based on the use of calculus was conducted by famous mathematicians Leonhard Euler and Carl Friedrich Gauss. Since that time, the rapidly emerging field of differential geometry has led to a deeper understanding of topics such as curvature and geodesy. Over time, a variety of approaches and techniques have been used in the research of curves and surfaces. These evolutions have contributed to a deeper understanding of science and technology. The surfaces corresponding to differential equations have recently attracted attention in a variety of multidisciplinary fields as well as in the geometrical analysis of differential equations. The time-dependent behavior of a curve or a surface is examined by its associated geometric flows in Euclidean spaces. If the arc-length of a curve (the first fundamental form of a surface) is preserved while it is evolving over time, then this curve (surface) is called inelastic [1]. The evolution of inelastic curves and flows on fluid surfaces can produce apparent motion, even in the absence of potential energy. For example, the oscillatory motion of a fixed-length cable or the flapping of paper in the wind can be modeled by inelastic geometric flows [1]. More explicitly, a surface evolution φ(s,u,t) for any time *t* is an isometric image of the original surface $\varphi \left(s,u,t_{0}\right)$ at any initial time *t*_0_. A developable surface φ(s,u,t) can be visualized as a physical phenomenon such as a fluctuating flag [1]. Furthermore, this circumstance naturally arises in a variety of applications in physics. For instance, the control of systems with excessive mobility (such as snake-like robots) was studied by Chirikjian and Burdick [2] and Mochiyama, Shimemura, and Kobayashi [3] from this perspective. Non-elastic curves and surface flows arise in computer graphics [4,5], computer animation [6], and even in many problems in mechanical motion science [2]. To explain what is commonly used in the above problems, there is a need to mathematically define evolutions that express non-elastic surfaces and curves. While there is much research in the literature about flow on planar curves, research on flow on surface evolutions is relatively infrequent [1]. In particular, the study of planar curve flows has been investigated using Gage, Hamilton [7], and Grayson’s [8] methods, where closed planar curves shrink to a point with the heat equation. Additionally, Gage investigated planar curve evolutions that preserve area [9]. Kwon and Park [10] previously provided a detailed analysis of the distinction between elastic and inelastic planar curve flows and their relation to the flow of the curve (motion) and fluid mechanics, and presented more advanced examples [1]. Kwon, Park, and Chi derived a general formula for elastic non-planar curves and developable surfaces based on the initial results on planar curves in *E*^3^ [1]. Firstly, they formulated the precise conditions via curvature and torsion for a curve on a surface to evolve via an elastic, non-planar flow. Then, they obtained corresponding evolutions for developable surfaces and showed that the set of solutions of partial differential equations characterizes elastic non-planar flows for space curves and developable surfaces. On the other hand, the partner-ruled surfaces were investigated by Li et al. using the Flc frame [11]. In addition, the partner-ruled surfaces formed by Darboux frame vectors defined on a regular surface were examined by Soukaina [12]. Also, the derivation of the modified Korteweg-de Vries equation from the motion of inextensible quaternionic curves was put forth by Eren [13]. In the context of curves, differential equations are often used to describe the relationship between a curve’s curvature and other geometric properties. Moreover, it is well-known that differential equations can also be used to describe the geometry and behavior of surfaces. Eren et al. examined partner-ruled surfaces with the alternative frame [14], quaternionic curves with the Frenet frame [15], and various problems in Minkowski space [16]. Moreover, Li et al. classified ruled surfaces according to their constant angles using polynomial curves with the Flc frame [17], and further investigated sweeping surfaces [18]. Recent studies have explored diverse aspects of differential geometry in Lorentzian and Minkowski settings, including the geometry and evolution of Hasimoto surfaces in Minkowski 3-space [19], the characterization of fixed-axis spacelike ruled surfaces and their evolute offsets [20], and the investigation of slant curves within Lorentzian doubly warped product manifolds [21]. Also, involute partner-ruled surfaces formed by involutes of spacelike curves in Minkowski three-space were investigated in [22]. The research on the generalized osculating-type ruled surfaces [23] and the generalized rectifying ruled surfaces of special singular curves expanded the ruled surface theory [24]. This research also aims to contribute to the literature on curves and surfaces. The study of simultaneous inextensible evolutions of partner-ruled surfaces has several real-life applications across science and engineering. In robotics and mechanism design, they offer a mathematical framework for analyzing the motion of linkages and robotic arms, where ruled surfaces naturally arise from the trajectories of moving elements. In the fields of computer graphics and animation, inextensible surface evolutions enable the creation of realistic simulations of surface motions without artificial stretching.

In this paper, we study the inextensible evolutions of the partner-ruled surfaces simultaneously constructed by a pair of vectors in the set of the Darboux and Frenet vectors of a unit parametrized space curve. We also present the invariants associated with these simultaneous partner-ruled surfaces and investigate the characterizations of their parameter curves. Several examples accompanied by graphical illustrations are provided.

## Basic concepts and findings

### Preliminaries on curves

Let us recall the basic concepts regarding the evolution of a moving curve in Euclidean 3-space *E*^3^ and provide the intrinsic equations involving its curvatures with respect to the moving Frenet frame.

Let α(s,t) be a moving space curve parameterized by arc length parameter *s* and evolved by time parameter *t*. The evolution is described by how *α* changes with *t* and $\frac{\partial \alpha }{\partial t}$ represents the velocity of deformation with respect to time, describing how the shape or position of the curve changes over time. The local geometric shape and also the evolution of the shape of the curve *α* are explicated via partial differentiations relative to *s* and *t*, respectively. Here and in the rest of the paper, *f*_*t*_, *f*_*s*_, *f*_*st*_, and *f*_*ts*_ denote $\frac{\partial f}{\partial t}$, $\frac{\partial f}{\partial s}$, $\frac{\partial^{2}f}{\partial s\partial t}$, and $\frac{\partial^{2}f}{\partial t\partial s}$ for any smooth *f*.

Accordingly, the partial differentiations of the elements of the Frenet frame {T,N,B} with respect to *s* and *t* are as follows:

$${\left[\begin{array}{c} T\\ N\\ B \end{array}\right]}_{s}=\left[\begin{array}{ccc} 0 & \kappa & 0\\ -\kappa & 0 & \tau \\ 0 & -\tau & 0 \end{array}\right]\left[\begin{array}{c} T\\ N\\ B \end{array}\right]$$ (1)

where κ is the curvature and *τ* is the torsion of the curve and


$${\left[\begin{array}{c} T\\ N\\ B \end{array}\right]}_{t}=\left[\begin{array}{ccc} 0 & \zeta_{1} & \zeta_{2}\\ -\zeta_{1} & 0 & \zeta_{3}\\ -\zeta_{2} & -\zeta_{3} & 0 \end{array}\right]\left[\begin{array}{c} T\\ N\\ B \end{array}\right],$$

where $\zeta_{i}$ is a continuously differentiable function depending on *s* and *t* for each  i∈{1,2,3}. Considering the compatibility condition $T_{st}=T_{ts},\;N_{st}=N_{ts}$, and $B_{st}=B_{ts}$, the following relationships are obtained as


$$\zeta_{1}=-\kappa \tau ,\;\;\zeta_{2}=\kappa_{s},\;\;\zeta_{3}=\frac{\kappa_{ss}-\kappa \tau^{2}}{\kappa }.$$

The curvatures of the evolving space curve satisfy the partial differential equations


$$\kappa_{t}=-2\kappa_{s}\tau -\kappa \tau_{s}\;\text{and}\;\tau_{t}=\kappa \kappa_{s}-2\tau \tau_{s}+{\left(\frac{\kappa_{ss}}{\kappa }\right)}_{s},[\text{1}].$$

Moreover, W=τT  +  κB is known as the Darboux vector describing the angular velocity of the Frenet frame along the curve. Thus, for the partial differential equations of the Darboux vector of the evolving space curve, we give the relations:


$$W_{t}={\left(\zeta_{3}\right)}_{s}T+\left(\zeta_{1}\tau -\zeta_{3}\kappa \right)N-\zeta_{2}\tau B\;\text{and}\;W_{s}=\tau_{s}T+\kappa_{s}B.$$

### Preliminaries on ruled surfaces

A surface formed by the motion of a straight line (directrix) along a given curve (base curve) is called a ruled surface, and it is parametrically represented by


P(s,u)=α(s)+uω(s)

with the base curve α(s) and directrix ω(s) [25]. The normal vector of P(s,u) is determined by

$$U=\frac{P_{s}\times P_{u}}{\left\|P_{s}\times P_{u}\right\|}$$ (2)

such that *P*_*s*_ and *P*_*u*_ are the tangent vectors of P(s,u).

Furthermore, the coefficients of the first fundamental form $I=Eds^{2}+2Fdsdu+Gdu^{2}$ of P(s,u) are introduced as

$$E=\left\langle P_{s},P_{s}\right\rangle ,\;\;F=\left\langle P_{s},P_{u}\right\rangle ,\;\;G=\left\langle P_{u},P_{u}\right\rangle .$$ (3)

The coefficients of the second fundamental form $II=eds^{2}+2fdsdu+gdu^{2}$ of P(s,u) are

$$e=\left\langle P_{ss},U\right\rangle ,\;\;f=\left\langle P_{su},U\right\rangle ,\;\;g=\left\langle P_{uu},U\right\rangle .$$ (4)

Additionally, the Gaussian and the mean curvatures of P(s,u) are given by

$$K=\frac{eg-f^{2}}{EG-F^{2}}\;\text{and}\;H=\frac{1}{2}\frac{Eg-2Ef+Ge}{EG-F^{2}},$$ (5)

respectively. In addition, the surfaces for which the Gaussian curvature vanishes are known as developable surfaces, and surfaces with zero mean curvature are referred to as minimal surfaces [25].

If the base curve α(s) and directrix ω(s) evolve by time parameter *t*, then the evolution of the ruled surface P(s,u) is represented by


P(s,u,t)=α(s,t)+uω(s,t).

A surface evolution P(s,u,t) and its flow *P*_*t*_ are said to be inextensible provided that the coefficients *E*, *F*, and *G* satisfy

$$E_{t}=F_{t}=G_{t}=0$$ (6)

for each time *t* [1]. This condition ensures that the first fundamental form (metric tensor) remains constant in time, i.e., the inextensibility of a surface evolution.

## Evolutions of partner-ruled surfaces with simultaneous inextensibility conditions

Two ruled surfaces are called partners if they share the same rulings. In this section, partner-ruled surfaces are simultaneously generated with a pair of vectors in Frenet–Darboux system along a generator curve, and their base curve and rulings are in one-to-one correspondence via the same parameter. The inextensible evolutions of partner-ruled surfaces are deformations of two surfaces that preserve the length of the tangent vectors along their generator curve. In other words, the surfaces may change their shapes, but they cannot simultaneously stretch or shrink along the generator curve. The partner-ruled surfaces are surfaces that are constructed by sweeping a space curve along another space curve using a specific set of vectors. Specifically, to construct evolutions of such pairs of surfaces, we use the evolving Darboux and Frenet vectors along a space curve evolving over time, and then we explore their simultaneous inextensible evolutions.

### Evolutions of *TN* partner-ruled surfaces

**Definition 1.** *Let* α(s,t) *be a moving space curve parameterized by arc length parameter s and evolved by time parameter t with the Frenet frame* {T,N,B}. *The two evolving ruled surfaces presented by*

$$\begin{array}{c} P_{N}^{T}\left(s,u,t\right)=T\left(s,t\right)+uN\left(s,t\right),\\ P_{T}^{N}\left(s,u,t\right)=N\left(s,t\right)+uT\left(s,t\right), \end{array}$$ (7)

*are termed as evolutions of the TN partner-ruled surfaces, which are associated with the tangent and principal normal vectors of the evolving generator curve α*.

**Theorem 1.** *The evolutions of the TN partner-ruled surfaces are simultaneously inextensible if the evolutions of the generator curve α are a circular helix for all values of time t.*

*Proof*: Let *α* be a circular helix for each *t*. Then its curvature and torsion are nonzero constants, i.e., $\kappa_{s}=0$ and $\tau_{s}=0$. These require $\kappa_{t}=-2\kappa_{s}\tau -\kappa \tau_{s}=0$ and $\tau_{t}=\kappa \kappa_{s}-2\tau \tau_{s}+{\left(\frac{\kappa_{ss}}{\kappa }\right)}_{s}=0.$ On the other hand, by considering Frenet formulae given in Eq (1) in the partial differentiations of Eq (7) with respect to *s* and *u*, the tangent vectors of the *TN* partner-ruled surfaces for each *t* are found as

$${\left(P_{N}^{T}\right)}_{s}=-u\kappa T\left(s,t\right)+\kappa N\left(s,t\right)+u\tau B\left(s,t\right),\;\;\;{\left(P_{N}^{T}\right)}_{u}=N\left(s,t\right),\;{\left(P_{T}^{N}\right)}_{s}=-\kappa T\left(s,t\right)+u\kappa N\left(s,t\right)+\tau B\left(s,t\right),\;\;\;{\left(P_{T}^{N}\right)}_{u}=T\left(s,t\right).\;$$ (8)

To determine the quantities given in Eq (3) for the *TN* partner-ruled surface, we compute the scalar products of the tangent vectors in Eq (8) with each other and we get

$$E_{TN}=\left(1+u^{2}\right)\kappa^{2}+u^{2}\tau^{2},\;\;F_{TN}=\kappa ,\;G_{TN}=1,\;E_{NT}=\left(1+u^{2}\right)\kappa^{2}+\tau^{2},\;\;F_{NT}=-\kappa ,\;G_{NT}=1.\;$$ (9)

Directly, the partial derivatives of these real-valued functions with respect to the time parameter are


$${\left(E_{TN}\right)}_{t}=2\kappa \kappa_{t}\left(1+u^{2}\right)+2\tau \tau_{t}u^{2},\;\;{\left(F_{TN}\right)}_{t}=\kappa_{t},\;{\left(\;G_{TN}\right)}_{t}=0$$

and


$${\left(E_{NT}\right)}_{t}=2\kappa \kappa_{t}\left(1+u^{2}\right)+2\tau \tau_{t},\;\;{\left(F_{NT}\right)}_{t}=-\kappa_{t},\;\;{\left(G_{NT}\right)}_{t}=0.$$

Finally, the facts of $\kappa_{t}=0$ and $\tau_{t}=0$ provide the condition outlined in (6), and the proof is concluded. □

**Theorem 2.** *The evolutions of the TN partner-ruled surfaces are simultaneously both developable and minimal if the evolutions of α are a straight line or a planar curve for all values of time t.*

*Proof*: As stated in (2), by the cross product of the tangent vectors given in Eq (8), we determine the normal vectors of the evolutions of the *TN* partner-ruled surfaces for each time *t* as follows:


$$U_{TN}=\frac{\tau N\left(s,t\right)-u\kappa B\left(s,t\right)}{\sqrt{u^{2}\kappa^{2}+\tau^{2}}}\;\text{and}\;U_{NT}=-\frac{\tau T\left(s,t\right)+\kappa B\left(s,t\right)}{\sqrt{\kappa^{2}+\tau^{2}}}.$$

The second order partial differentiations of Eq (8) with respect to *s* and *u* are


$${\left(P_{N}^{T}\right)}_{ss}=-\kappa \left(\kappa +u\kappa_{s}\right)T\left(s,t\right)+\left(\kappa_{s}-u\kappa^{2}\right)N\left(s,t\right)+\left(\tau \left(\kappa -u\tau \right)+u\tau_{s}\right)B\left(s,t\right),\;\;\;{\left(P_{N}^{T}\right)}_{su}=-\kappa T\left(s,t\right)+\tau B\left(s,t\right),\;\;\;{\left(P_{N}^{T}\right)}_{uu}=0,\;$$

and


$${\left(P_{T}^{N}\right)}_{ss}=-\left(u\kappa^{2}+\kappa_{s}\right)T\left(s,t\right)+\left(u\kappa_{s}-\kappa^{2}\right)N\left(s,t\right)+\left(\tau \left(u\kappa -\tau \right)+\tau_{s}\right)B\left(s,t\right),\;\;{\left(P_{T}^{N}\right)}_{su}=\kappa N\left(s,t\right),\;\;\;{\left(P_{T}^{N}\right)}_{uu}\;=0.\;$$

As stated in Eq (4), the scalar products of each vector in the last equations with the normal vector of the evolutions of the *TN* partner-ruled surfaces for each *t*, the second fundamental form coefficients are found as

$$e_{TN}=\frac{u\left(\tau \kappa_{s}+\kappa \left(\tau^{2}-\tau_{s}\right)\right)}{\sqrt{\kappa^{2}+\tau^{2}}},\;\;f_{TN}=0,\;\;g_{TN}=0,\;e_{NT}=\frac{-\left(1+u^{2}\right)\kappa^{2}\tau +u\tau \kappa_{s}+u\kappa \left(\tau^{2}-\tau_{s}\right)}{\sqrt{u^{2}\kappa^{2}+\tau^{2}}},\;\;f_{NT}=\frac{\kappa \tau }{\sqrt{u^{2}\kappa^{2}+\tau^{2}}},\;\;g_{NT}=0.\;$$ (10)

Therefore, considering Eqs (9) together with (10) in Eq (5) give the Gaussian and mean curvature of the evolutions of the *TN* partner-ruled surfaces at each *t* as


$$K_{TN}=0,\;\;\;H_{TN}=\frac{\tau \kappa_{s}+\kappa \left(\tau^{2}-\tau_{s}\right)}{2u{\left(\kappa^{2}+\tau^{2}\right)}^{3/2}}$$

and


$$K_{NT}=-\frac{\kappa^{2}\tau^{2}}{{\left(u^{2}\kappa^{2}+\tau^{2}\right)}^{2}},H_{NT}=\frac{\left(1-u^{2}\right)\kappa^{2}\tau +u\tau \kappa_{s}+u\kappa \left(\tau^{2}-\tau_{s}\right)}{2{\left(u^{2}\kappa^{2}+\tau^{2}\right)}^{3/2}}.$$

If *α* is a straight line or planar, then κ=0 or τ=0. Any of these conditions requires $K_{TN}=K_{NT}=0$ and $H_{TN}=H_{NT}=0$, so that these evolutions of surfaces are simultaneously developable and minimal. □

**theorem 3.** *The s–parameter curves of the TN partner-ruled surfaces* $P_{N}^{T}\left(s,u\right)$ *and* $P_{T}^{N}\left(s,u\right)$ *are simultaneously asymptotic if the generator curve α is planar, but they are not simultaneously geodesic.*

*Proof*: The scalar products of the second partial derivatives and the normal vectors of the *TN*–partner-ruled surfaces are computed as follows:


$$\left\langle {\left(P_{N}^{T}\right)}_{ss},U_{N}^{T}\right\rangle =\frac{u\left(\tau \kappa_{s}+\kappa \left(\tau^{2}-\tau_{s}\right)\right)}{\sqrt{\kappa^{2}+\tau^{2}}}$$

and


$$\left\langle {\left(P_{T}^{N}\right)}_{ss},U_{T}^{N}\right\rangle =\frac{-\left(1+u^{2}\right)\kappa^{2}\tau +u\tau \kappa_{s}+u\kappa \left(\tau^{2}-\tau_{s}\right)}{\sqrt{u^{2}\kappa^{2}+\tau^{2}}}.$$

From here, if the generator curve of the *TN* partner-ruled surfaces is planar, i.e., τ=0, then $\left\langle {\left(P_{N}^{T}\right)}_{ss},U_{N}^{T}\right\rangle =0$ and $\left\langle {\left(P_{T}^{N}\right)}_{ss},U_{T}^{N}\right\rangle =0$ are satisfied, which means that the *s*–parameter curves of them are simultaneously asymptotic. On the contrary, they are simultaneously non-geodesic, because ${\left(P_{N}^{T}\right)}_{ss}\times U_{N}^{T}\neq 0$ and ${\left(P_{T}^{N}\right)}_{ss}\times U_{T}^{N}\neq 0$ since κ and *τ* cannot vanish together in the equalities


$${\left(P_{N}^{T}\right)}_{ss}\times U_{N}^{T}=\frac{u\kappa^{3}-\kappa \kappa_{s}}{\sqrt{\kappa^{2}+\tau^{2}}}T\left(s,t\right)-\frac{\kappa^{3}+\kappa \left(\tau^{2}+u\kappa_{s}\right)+u\tau \left(-\tau^{2}+\tau_{s}\right)}{\sqrt{\kappa^{2}+\tau^{2}}}N\left(s,t\right)+\frac{\tau \left(-u\kappa^{2}+\kappa_{s}\right)}{\sqrt{\kappa^{2}+\tau^{2}}}B\left(s,t\right)$$

and


$${\left(P_{T}^{N}\right)}_{ss}\times U_{T}^{N}=\frac{u\kappa^{3}+\tau^{3}-u\kappa \left(\tau^{2}+u\kappa_{s}\right)-\tau \tau_{s}}{\sqrt{u^{2}\kappa^{2}+\tau^{2}}}T\left(s,t\right)-\frac{u\kappa \left(u\kappa^{2}+\kappa_{s}\right)}{\sqrt{u^{2}\kappa^{2}+\tau^{2}}}N\left(s,t\right)-\frac{\tau \left(u\kappa^{2}+\kappa_{s}\right)}{\sqrt{u^{2}\kappa^{2}+\tau^{2}}}B\left(s,t\right).$$

□

**theorem 4.** *The u–parameter curves of the TN partner-ruled surfaces are both simultaneously geodesic and asymptotic.*

*Proof*: Since ${\left(P_{N}^{T}\right)}_{uu}$ and ${\left(P_{T}^{N}\right)}_{uu}$ are zero vectors, it is obvious that ${\left(P_{N}^{T}\right)}_{uu}\times U_{N}^{T}=0$, ${\left(P_{T}^{N}\right)}_{uu}\times U_{T}^{N}=0$, $\left\langle {\left(P_{N}^{T}\right)}_{uu},U_{N}^{T}\right\rangle =0$, and $\left\langle {\left(P_{T}^{N}\right)}_{uu},U_{T}^{N}\right\rangle =0$. So, these directly prove that the *u*–parameter curves of the *TN* partner-ruled surfaces simultaneously are geodesic and asymptotic. □

### Evolutions of *TB* partner-ruled surfaces

**Definition 2.** *Let* α(s,t) *be a moving space curve parameterized by arc-length parameter s and evolved by time parameter t with the Frenet frame* {T,N,B}. *The two evolving ruled surfaces over time t are presented by*

$$\left\{ \begin{array}{c} P_{B}^{T}(s,u,t)=T(s,t)+uB(s,t),\\ P_{T}^{B}(s,u,t)=B(s,t)+uT(s,t), \end{array}\right.$$ (11)

*are called evolutions of the TB partner-ruled surfaces associated with the tangent and binormal vectors along the evolving generator curve α.*

**Theorem 5.** *Let* $P_{B}^{T}$ *and* $P_{T}^{B}$ *represent the evolutions of the TB partner-ruled surfaces, then they are simultaneously inextensible if their generator curve is a circular helix for all values of time t.*

*Proof*: By using the Frenet derivative formulas in the partial differentiations of Eq (11) with respect to *s* and *u*, we achieve that

$${\left(P_{B}^{T}\right)}_{s}=\kappa N\left(s,t\right)-u\tau B\left(s,t\right),\;\;{\left(P_{B}^{T}\right)}_{u}=B\left(s,t\right),\;{\left(P_{T}^{B}\right)}_{u}=u\kappa N\left(s,t\right)-\tau B\left(s,t\right),\;\;{\left(P_{T}^{B}\right)}_{u}=T\left(s,t\right).\;$$ (12)

Using Eq (12) in Eq (2), the coefficients for the evolutions of the *TB* partner-ruled surfaces are obtained as

$$E_{TB}=\kappa^{2}+u^{2}\tau^{2},\;\;F_{TB}=-u\tau ,\;G_{TB}=1,\;E_{BT}=u^{2}\kappa^{2}+\tau^{2},\;\;F_{BT}=0,\;G_{BT}=1.\;$$ (13)

Then the partial derivatives of these coefficients with respect to the time parameter are


$${\left(E_{TB}\right)}_{t}=2\kappa \kappa_{t}+2\tau \tau_{t}u^{2},\;\;{\left(F_{TB}\right)}_{t}=-\tau_{t}u,\;\;{\left(G_{TB}\right)}_{t}=0$$

and


$${\left(E_{BT}\right)}_{t}=\kappa_{t}u^{2}+2\tau \tau_{t},\;\;{\left(F_{BT}\right)}_{t}=0,\;\;{\left(G_{BT}\right)}_{t}=0.$$

Under the assumption of the evolution of the generator curve being a circular helix for all values of time *t*, then $\kappa_{s}=0$ and $\tau_{s}=0$, which requires $\kappa_{t}=0$ and $\tau_{t}=0$. These satisfy the condition in (6) and complete the proof. □

**theorem 6.** *The TB partner-ruled surfaces are simultaneously developable if and only if the generator curve of these surfaces is a straight line or planar, but they are not simultaneously minimal.*

*Proof*: The normal vectors of the *TB* partner-ruled surfaces are computed as follows:


$$U_{TB}=T\left(s,t\right)\;\text{and}\;U_{BT}=-\frac{\tau N\left(s,t\right)+u\kappa B\left(s,t\right)}{\sqrt{u^{2}\kappa^{2}+\tau^{2}}}.$$

Differentiating Eq (12) for *s* and *u*, we get


$${\left(P_{B}^{T}\right)}_{ss}=-\kappa^{2}T\left(s,t\right)+\kappa_{s}N\left(s,t\right)+\left(\tau \left(\kappa +u\tau \right)-u\tau_{s}\right)B\left(s,t\right),\;{\left(P_{B}^{T}\right)}_{su}=-\tau B\left(s,t\right),\;\;\;{\left(P_{B}^{T}\right)}_{uu}=0,\;$$

and


$${\left(P_{T}^{B}\right)}_{ss}=-u\kappa^{2}T\left(s,t\right)+u\kappa_{s}N\left(s,t\right)+\left(u\kappa \tau +\tau^{2}-\tau_{s}\right)B\left(s,t\right),\;\;{\left(P_{T}^{B}\right)}_{su}=\kappa N\left(s,t\right),\;\;\;\;{\left(P_{T}^{B}\right)}_{uu}=0.\;$$

These equalities allow us to determine the second fundamental form coefficients of the *TB* partner-ruled surfaces as

$$e_{TB}=-\kappa^{2},\;\;f_{TB}=0,\;\;g_{TB}=0.\;e_{BT}=\frac{-u\tau \left(\kappa \left(u\kappa +\tau \right)+\kappa_{s}\right)+u\kappa \tau_{s}}{\sqrt{u^{2}\kappa^{2}+\tau^{2}}},\;\;f_{BT}=-\frac{\kappa \tau }{\sqrt{u^{2}\kappa^{2}+\tau^{2}}},\;\;g_{BT}=0.\;$$ (14)

Therefore, the Gaussian and mean curvatures of the *TB* partner-ruled surface are determined by substituting Eqs (13), (14) into Eq (5) as follows:


$$K_{TB}=0,\;\;\;H_{TB}=-\frac{1}{2}$$

and


$$K_{BT}=-\frac{\kappa^{2}\tau^{2}}{{\left(u^{2}\kappa^{2}+\tau^{2}\right)}^{2}},H_{BT}=\frac{-u\tau \left(\kappa \left(u\kappa +\tau \right)+\kappa_{s}\right)+u\kappa \tau_{s}}{2{\left(u^{2}\kappa^{2}+\tau^{2}\right)}^{3/2}}.$$

From here, κ=0 or τ=0 iff $K_{TB}=K_{BT}=0$. However, the *TB* partner-ruled surfaces are not simultaneously minimal since $H_{TB}\neq 0$. □

**Theorem 7.** *The s–parameter curves of the TB partner-ruled surfaces* $P_{B}^{T}$ *and* $P_{T}^{B}$ *are simultaneously asymptotic if the generator curve of these surfaces is a straight line. However, they are not simultaneously geodesics.*

*Proof*: By direct calculations, we get


$$\left\langle {\left(P_{B}^{T}\right)}_{ss},U_{B}^{T}\right\rangle =-\kappa^{2}\;\text{and}\;\left\langle {\left(P_{T}^{B}\right)}_{ss},U_{T}^{B}\right\rangle =\frac{-u\tau \left(\kappa \left(u\kappa +\tau \right)+\kappa_{s}\right)+u\kappa \tau_{s}}{\sqrt{u^{2}\kappa^{2}+\tau^{2}}}.$$

The assumption of κ=0 proves the *s*–parameter curves to be simultaneously asymptotic. However, from the cross products


$${\left(P_{B}^{T}\right)}_{ss}\times U_{B}^{T}=\left(\tau \left(\kappa +u\tau \right)-u\tau_{s}\right)N\left(s,t\right)-\kappa_{s}B\left(s,t\right),$$

and


$${\left(P_{T}^{B}\right)}_{ss}\times U_{T}^{B}=\frac{\tau^{3}+u\kappa \left(\tau^{2}-u\kappa_{s}\right)-\tau \tau_{s}}{\sqrt{u^{2}\kappa^{2}+\tau^{2}}}T\left(s,t\right)-\frac{u^{2}\kappa^{3}}{\sqrt{u^{2}\kappa^{2}+\tau^{2}}}N\left(s,t\right)+\frac{u\kappa^{2}\tau }{\sqrt{u^{2}\kappa^{2}+\tau^{2}}}B\left(s,t\right).$$

It is obvious that the *s*–parameter curves of the *TB* partner-ruled surfaces are not simultaneously geodesic since $\frac{\partial^{2}P_{B}^{T}}{\partial s^{2}}\times U_{B}^{T}\neq 0$ and $\frac{\partial^{2}P_{T}^{B}}{\partial s^{2}}\times U_{T}^{B}\neq 0$. □

**Theorem 8.** *Let* $P_{B}^{T}$ *and* $P_{T}^{B}$ *represent the TB partner-ruled surfaces, then the u–parameter curves of the TB partner-ruled surfaces are simultaneously geodesic and asymptotic.*

*Proof*: From the facts that ${\left(P_{B}^{T}\right)}_{uu}=0$ and ${\left(P_{T}^{B}\right)}_{uu}=0$ the proof is trivial. □

### Evolutions of *NB* partner-ruled surfaces

**Definition 3.** *Let* α(s,t) *be a moving space curve parameterized by arc length parameter s and evolved by time parameter t with the Frenet frame* {T,N,B}. *The two evolving ruled surfaces over time t are presented by*

$$\{\begin{array}{c} P_{B}^{N}\left(s,u,t\right)=N\left(s,t\right)+uB\left(s,t\right),\\ P_{N}^{B}\left(s,u,t\right)=B\left(s,t\right)+uN\left(s,t\right), \end{array}$$ (15)

*are called evolutions of the NB partner-ruled surfaces associated with the principal normal and binormal vectors of the generator curve α.*

**Theorem 9.** *The evolutions of the NB partner-ruled surfaces are simultaneously inextensible if their evolving generator curve is a circular helix for all values of time t.*

*Proof*: The tangent vector and thus the coefficients of the first fundamental forms of the *NB* partner-ruled surfaces are found as

$${\left(P_{B}^{N}\right)}_{s}=-\kappa T\left(s,t\right)+\left(\tau -u\tau \right)B\left(s,t\right),\;\;{\left(P_{B}^{N}\right)}_{u}=B\left(s,t\right),\;{\left(P_{N}^{B}\right)}_{s}=-u\kappa T\left(s,t\right)-\left(\tau -u\tau \right)B\left(s,t\right),\;\;{\left(P_{N}^{B}\right)}_{u}=N\left(s,t\right),\;$$ (16)

and

$$E_{NB}=\kappa^{2}+{\left(-1+u\right)}^{2}\tau^{2},\;\;F_{NB}=-\left(-1+u\right)\tau ,\;G_{NB}=1,\;E_{BN}=u^{2}\kappa^{2}+{\left(-1+u\right)}^{2}\tau^{2},\;\;F_{BN}=0,\;G_{BN}=1,\;$$ (17)

respectively. Taking the partial derivatives of Eq (17) for time parameter *t*, we get


$${\left(E_{NB}\right)}_{t}=2\kappa \kappa_{t}+2\tau \tau_{t}{\left(-1+u\right)}^{2},\;\;{\left(F_{NB}\right)}_{t}=\tau_{t}\left(1-u\right),\;\;{\left(G_{NB}\right)}_{t}=0$$

and


$${\left(E_{BN}\right)}_{t}=2\kappa \kappa_{t}u^{2}+2\tau \tau_{t}{\left(-1+u\right)}^{2},\;\;{\left(F_{BN}\right)}_{t}=0,\;\;{\left(G_{BN}\right)}_{t}=0.$$

Provided that the generator curve is a circular helix for all values of time *t*, then $\kappa_{t}=0$ and $\tau_{t}=0$. Therefore, the condition stated in (6) holds, and the proof is thus completed. □

**Theorem 10.** *Let* $P_{B}^{N}$ *and* $P_{N}^{B}$ *denote the NB partner-ruled surfaces with the Frenet frame* {T,N,B}. *Then, the NB partner-ruled surfaces are simultaneously developable if and only if the generator curve of these surfaces is a straight line or planar, but they are not simultaneously minimal.*

*Proof*: The normal vectors of the partner-ruled surfaces are computed as


$$U_{NB}=N\left(s,t\right)\;\text{and}\;U_{BN}=-\frac{\tau \left(u-1\right)T\left(s,t\right)+u\kappa B\left(s,t\right)}{\sqrt{u^{2}\kappa^{2}+{\left(u-1\right)}^{2}\tau^{2}}}.$$

Differentiations of Eq (16) are


$${\left(P_{B}^{N}\right)}_{ss}=-\kappa_{s}T\left(s,t\right)-\kappa^{2}N\left(s,t\right)+\left(u-1\right)\left(\tau^{2}-\tau_{s}\right)B\left(s,t\right),\;\;\;{\left(P_{B}^{N}\right)}_{su}=-\tau B\left(s,t\right),\;\;\;{\left(P_{B}^{N}\right)}_{uu}=0,\;$$

and


$${\left(P_{N}^{B}\right)}_{ss}=-u\kappa_{s}T\left(s,t\right)-u\kappa^{2}N\left(s,t\right)+\left(u-1\right)\left(\tau^{2}-\tau_{s}\right)B\left(s,t\right),\;{\left(P_{N}^{B}\right)}_{su}=-\kappa T\left(s,t\right)+\tau B\left(s,t\right),\;\;\;{\left(P_{N}^{B}\right)}_{uu}\;=0.\;$$

Then the coefficients of the second fundamental form of the *NB* partner-ruled surfaces are obtained as

$$e_{NB}=-\kappa^{2},\;\;f_{NB}=0,\;\;g_{NB}=0,\;e_{BN}=\frac{\left(u-1\right)u\left(\tau \kappa_{s}+\kappa \left(\tau^{2}-\tau_{s}\right)\right)}{\sqrt{u^{2}\kappa^{2}+{\left(u-1\right)}^{2}\tau^{2}}},\;\;f_{BN}=-\frac{\kappa \tau }{\sqrt{u^{2}\kappa^{2}+{\left(u-1\right)}^{2}\tau^{2}}},\;\;g_{BN}=0.\;$$ (18)

Therefore, by putting Eqs (17) and (18) in Eq (5), the Gaussian curvatures $K_{NB},\;K_{BN}$ and the mean curvatures $H_{NB},\;H_{BN}$ of the partner-ruled surfaces are determined as follows:


$$K_{NB}=0,\;\;\;H_{NB}=-\frac{1}{2}$$

and


$$K_{BN}=-\frac{\kappa^{2}\tau^{2}}{{\left(u^{2}\kappa^{2}+{\left(u-1\right)}^{2}\tau^{2}\right)}^{2}},H_{BN}=\frac{\left(u-1\right)u\left(\tau \kappa_{s}+\kappa \left(\tau^{2}-\tau_{s}\right)\right)}{2{\left(u^{2}\kappa^{2}+{\left(u-1\right)}^{2}\tau^{2}\right)}^{3/2}}.$$

From these last equalities, κ=0 or τ=0 if and only if $K_{NB}=K_{BN}=0$. On the other hand, since $H_{NB}\neq 0$, it can be concluded that the *NB* partner-ruled surfaces are not simultaneously minimal. □

**Theorem 11.** *The s–parameter curves of the NB partner-ruled surfaces are simultaneously asymptotic if the generator curve of these surfaces is a straight line. However, they are not simultaneously geodesics.*

*Proof*: The proof is carried out in a similar manner to the proof of the theorem given for the *TN* partner-ruled surfaces. □

**Theorem 12.** *Let* $P_{B}^{N}$ *and* $P_{N}^{B}$ *be NB partner-ruled surfaces, then the u–parameter curves of these surfaces are simultaneously geodesic and asymptotic.*

*Proof*: ${\left(P_{B}^{N}\right)}_{uu}=0$ and ${\left(P_{N}^{B}\right)}_{uu}=0$, and the proof is trivial. □

### Evolutions of *NW* partner-ruled surfaces

**Definition 4.** *Let* α(s,t) *be a moving space curve parameterized by arc length parameter s and evolved by time parameter t with Darboux vector* W=τT  +  κB *and the Frenet frame* {T,N,B}. *The two evolving ruled surfaces over time t presented by*

$$\{\begin{array}{c} P_{W}^{N}\left(s,u,t\right)=N\left(s,t\right)+uW\left(s,t\right),\\ P_{N}^{W}\left(s,u,t\right)=W\left(s,t\right)+uN\left(s,t\right), \end{array}$$ (19)

*are said to be evolutions of the NW partner-ruled surfaces associated with the principal normal vector N and the Darboux vector W along α for each t.*

**Theorem 13.** *Let* $P_{W}^{N}$ *and* $P_{N}^{W}$ *represent the NW partner-ruled surfaces. Then the evolutions of these surfaces are simultaneously inextensible if their evolving generator curve is a circular helix for all values of time t.*

*Proof*: By differentiating the surfaces given by Eq (19) and using Frenet frame derivative formulae, one achieves

$${\left(P_{W}^{N}\right)}_{s}=\left(u\tau_{s}-\kappa \right)T\left(s,t\right)+u\kappa \tau N\left(s,t\right)+\left(\tau -u\kappa \tau +u\kappa_{s}\right)B\left(s,t\right),\;\;{\left(P_{W}^{N}\right)}_{u}=\tau T\left(s,t\right)+\kappa B\left(s,t\right),\;{\left(P_{N}^{W}\right)}_{s}=\left(\tau_{s}-u\kappa \right)T\left(s,t\right)+\kappa \tau N\left(s,t\right)+\left(u\tau -\kappa \tau +\kappa_{s}\right)B\left(s,t\right),\;\;{\left(P_{N}^{W}\right)}_{u}=N\left(s,t\right).\;$$ (20)

By taking the cross product of the first partial derivatives from Eq (20) and using Eq (2), the normal vectors of the *NW* partner-ruled surfaces become

$$E_{NW}=u^{2}\kappa^{2}\tau^{2}+{\left(u\kappa_{s}-u\kappa \tau +\tau \right)}^{2}+{\left(\kappa -u\tau_{s}\right)}^{2},\;F_{NW}=u\left({\left(\kappa \tau \right)}_{s}-\kappa^{2}\tau \right),\;G_{NW}=\kappa^{2}+\tau^{2},\;E_{WN}=\kappa^{2}\tau^{2}+{\left(u\tau -\kappa \tau +\kappa_{s}\right)}^{2}+{\left(\tau_{s}-u\kappa \right)}^{2},\;\;F_{WN}=\kappa \tau ,\;G_{WN}=1.\;$$ (21)

Taking the partial derivatives of Eq (21) for *t*, we get


$${\left(E_{NW}\right)}_{t}=2\left(\kappa \kappa_{t}+\tau \tau_{t}+u\left(\kappa_{s}\tau_{t}-\kappa_{t}\tau_{s}-\kappa \tau_{st}+\kappa_{st}\tau -\kappa_{t}\tau^{2}-2\kappa \tau \tau_{t}\right)\;-u^{2}\left(\kappa_{s}\kappa_{st}+\tau_{s}\tau_{st}-\kappa \kappa_{s}\tau_{t}-\kappa \kappa_{st}\tau -\kappa_{s}\kappa_{t}\tau +2\kappa^{2}\tau \tau_{t}+2\kappa \kappa_{t}\tau^{2}\right)\;\right),\;\;\;{\left(F_{NW}\right)}_{t}=u\left(\kappa_{s}\kappa_{t}-\kappa^{2}\tau_{t}+\tau_{s}\tau_{t}-2\kappa \kappa_{t}\tau +\kappa \kappa_{st}+\tau \tau_{st}\right),\;\;\;{\left(G_{NW}\right)}_{t}=2\left(\kappa \kappa_{t}+\tau \tau_{t}\right)\;$$

and


$$\frac{\partial E_{WN}}{\partial t}=2\left(2\kappa^{2}\tau \tau_{t}-\kappa \kappa_{s}\tau_{t}-\kappa \kappa_{st}\tau +2\kappa \kappa_{t}\tau^{2}+\kappa_{s}\kappa_{st}-\kappa_{s}\kappa_{t}\tau +\tau_{s}\tau_{st}\;+u\left(\kappa_{s}\tau_{t}+\kappa_{st}\tau -\kappa_{t}\tau^{2}-\kappa_{t}\tau_{s}-\kappa \tau_{st}-2\kappa \tau \tau_{t}\right)+u^{2}\left(\kappa \kappa_{t}+\tau \tau_{t}\right)\;\right),\;\;\;\frac{\partial F_{WN}}{\partial t}=\tau \kappa_{t}+\kappa \tau_{t},\;\;\;\frac{\partial G_{WN}}{\partial t}=0.\;$$

It can be seen that if the κ and *τ* are constants, which means $\kappa_{s}=\tau_{s}=0$, i.e., $\kappa_{t}=\tau_{t}=0$, then the condition specified in (6) is satisfied, and thus the proof is completed. □

**Theorem 14.** *Let* $P_{W}^{N}$ *and* $P_{N}^{W}$ *be NW partner-ruled surfaces associated with the principal normal vector N and the Darboux vector W, then the NW partner-ruled surfaces are simultaneously non-minimal and developable if* κ *is a nonzero constant and* τ=0.

*Proof*: Utilizing Eqs (2) and (21), the normal vectors of the corresponding the *NW* partner-ruled surfaces are determined by


$$U_{NW}=\left(\frac{u\kappa^{2}\tau }{\sqrt{u^{2}\kappa^{4}\tau^{2}+u^{2}\kappa^{2}\tau^{4}+{\left(\kappa^{2}+\tau^{2}+u\left(\kappa_{s}\tau -\kappa \tau_{s}-\kappa \tau^{2}\right)\right)}^{2}}}\right)T\left(s,t\right)\;\;\;\;\;\;\;\;\;\;\;\;\;\;\;\;\;\;\;\;\;+\left(\frac{\kappa^{2}+\tau^{2}+u\left(\kappa_{s}\tau -\kappa \tau_{s}-\kappa \tau^{2}\right)}{\sqrt{u^{2}\kappa^{4}\tau^{2}+u^{2}\kappa^{2}\tau^{4}+{\left(\kappa^{2}+\tau^{2}+u\left(\kappa_{s}\tau -\kappa \tau_{s}-\kappa \tau^{2}\right)\right)}^{2}}}\right)N\left(s,t\right)\;\;\;\;\;\;\;\;\;\;\;\;\;\;\;\;\;\;\;\;\;\;-\left(\frac{u\kappa \tau^{2}}{\sqrt{u^{2}\kappa^{4}\tau^{2}+u^{2}\kappa^{2}\tau^{4}+{\left(\kappa^{2}+\tau^{2}+u\left(\kappa_{s}\tau -\kappa \tau_{s}-\kappa \tau^{2}\right)\right)}^{2}}}\right)B\left(s,t\right)\;$$

and


$$U_{WN}=\left(\frac{\kappa \tau -\kappa_{s}-u\tau }{\sqrt{{\left(\kappa \tau -\kappa_{s}-u\tau \right)}^{2}+{\left(\tau_{s}-u\kappa \right)}^{2}}}\right)T\left(s,t\right)+\left(\frac{\tau_{s}-u\kappa }{\sqrt{{\left(\kappa \tau -\kappa_{s}-u\tau \right)}^{2}+{\left(\tau_{s}-u\kappa \right)}^{2}}}\right)B\left(s,t\right)$$

By differentiating Eq (20), it is found as


$$\begin{array}{c} {\left(P_{W}^{N}\right)}_{ss}=\left(-\kappa_{s}+u\left(\tau_{ss}-\kappa^{2}\tau \right)\right)T\left(s,t\right)+\left(-\kappa^{2}+u\left(\tau \kappa_{s}+2\kappa \tau_{s}\right)\right)N\left(s,t\right)\\ \;\;\;+\left(\tau_{s}-\tau^{2}-u\left(2\kappa \tau^{2}-2\tau \kappa_{s}+\kappa \tau_{s}+\kappa_{ss}\right)\right)B\left(s,t\right),\\ {\left(P_{W}^{N}\right)}_{su}=\tau_{s}T\left(s,t\right)+\kappa \tau N\left(s,t\right)+\left(\kappa_{s}-\kappa \tau \right)B\left(s,t\right),\\ {\left(P_{W}^{N}\right)}_{uu}=0, \end{array}$$

and


$$\begin{array}{c} {\left(P_{N}^{W}\right)}_{ss}=\left(\tau_{ss}-\kappa^{2}\tau -u\kappa_{s}\right)T\left(s,t\right)+\left(\tau \kappa_{s}+2\kappa \tau_{s}-u\kappa^{2}\right)N\left(s,t\right)\\ \;\;\;+\left(\left(u-\kappa \right)\tau_{s}+\kappa_{ss}-\tau \left(\left(u-2\kappa \right)\tau +2\kappa_{s}\right)\right)B\left(s,t\right),\\ {\left(P_{N}^{W}\right)}_{su}=-\kappa T\left(s,t\right)+\tau B\left(s,t\right)\\ {\left(P_{N}^{W}\right)}_{uu}\;=0. \end{array}$$

The coefficients of the second fundamental form of the *NW* partner-ruled surfaces are obtained as

$$e_{NW}=\frac{\left(\kappa^{4}\tau^{2}u^{2}-\kappa^{4}+\kappa^{3}\tau^{2}u+3\kappa^{3}\tau_{s}u-\kappa^{2}\kappa_{s}\tau u-2\kappa^{2}\tau^{4}u^{2}-\kappa^{2}\tau^{2}\tau_{s}u^{2}-\kappa^{2}\tau^{2}+\kappa^{2}\tau \tau_{ss}u^{2}\;-2\kappa^{2}\tau_{s}^{2}u^{2}+\kappa \kappa_{s}\tau^{3}u^{2}+\kappa \kappa_{s}\tau \tau_{s}u^{2}-\kappa \kappa_{ss}\tau^{2}u^{2}+\kappa \tau^{4}u+\kappa \tau^{2}\tau_{s}u+\kappa_{s}^{2}\tau^{2}u^{2}+\kappa_{s}\tau^{3}u\;\right)}{\sqrt{u^{2}\kappa^{4}\tau^{2}+u^{2}\kappa^{2}\tau^{4}+{\left(\kappa^{2}+\tau \left(\tau +u\kappa_{s}\right)-u\kappa \left(\tau^{2}+\tau_{s}\right)\right)}^{2}}}\;\;f_{NW}=\frac{\kappa^{3}\tau +\kappa \tau^{3}}{\sqrt{u^{2}\kappa^{4}\tau^{2}+u^{2}\kappa^{2}\tau^{4}+{\left(\kappa^{2}+\tau \left(\tau +u\kappa_{s}\right)-u\kappa \left(\tau^{2}+\tau_{s}\right)\right)}^{2}}},\;\;g_{NW}=0,\;\;e_{WN}=\frac{\left(\left(\tau_{s}-u\kappa \right)\left(\left(u-\kappa \right)\tau_{s}+\kappa_{ss}-\tau \left(\left(u-2\kappa \right)\tau +2\kappa_{s}\right)\right)+\left(\left(u-\kappa \right)\tau +\kappa_{s}\right)\left(\kappa^{2}\tau +u\kappa_{s}-\tau_{ss}\right)\right)}{\sqrt{{\left(\left(u-\kappa \right)\tau +\kappa_{s}\right)}^{2}+{\left(\tau_{s}-u\kappa \right)}^{2}}},\;\;\;f_{WN}=\frac{-\kappa^{2}\tau +\kappa \kappa_{s}+\tau \tau_{s}}{\sqrt{{\left(\left(u-\kappa \right)\tau +\kappa_{s}\right)}^{2}+{\left(\tau_{s}-u\kappa \right)}^{2}}},\;\;g_{WN}=0.\;$$ (22)

Therefore, by substituting Eqs (21) and (22) into Eq (5), the Gaussian curvatures $K_{NW},\;K_{WN}$ and the mean curvatures $H_{NW},\;H_{WN}$ of the partner-ruled surfaces are determined as follows:


$$K_{NW}=\frac{-\kappa^{2}\tau^{2}{\left(\kappa^{2}+\tau^{2}\right)}^{2}}{{\left(\tau^{2}{\left(\tau +u\kappa_{s}\right)}^{2}-2u\kappa^{3}\left(\tau^{2}+\tau_{s}\right)-2u\kappa \tau \left(\tau +u\kappa_{s}\right)\left(\tau^{2}+\tau_{s}\right)\kappa^{4}\left(1+u^{2}\tau^{2}\right)\;+\kappa^{2}\left(2u^{2}\tau^{4}+2u\tau \kappa_{s}+u^{2}{\tau_{s}}^{2}+2\tau^{2}\left(1+u^{2}\tau_{s}\right)\right)\;\right)}^{2}},\;\;\;\;\;H_{NW}=-\frac{\left(\kappa^{2}+\tau^{2}\right)\left(\kappa^{4}\left(1+u^{2}\tau^{2}\right)+\kappa^{2}\left(2u^{2}\tau^{4}+2u^{2}{\tau_{s}}^{2}+\tau^{2}\left(1+u^{2}\tau_{s}\right)+u\tau \left(3\kappa_{s}-u\tau_{ss}\right)\right)\;-u\tau^{2}\kappa_{s}\left(\tau +u\kappa_{s}\right)-3u\kappa^{3}\left(\tau^{2}+\tau_{s}\right)-u\kappa \tau \left(\tau^{3}+u\tau^{2}\kappa_{s}+u\kappa_{s}\tau_{s}-\tau \left(\tau_{s}+u\kappa_{ss}\right)\right)\;\right)}{2{\left(u^{2}\kappa^{4}\tau^{2}+u^{2}\kappa^{2}\tau^{4}+{\left(\kappa^{2}+\tau \left(\tau +u\kappa_{s}\right)-u\kappa \left(\tau^{2}+\tau_{s}\right)\right)}^{2}\right)}^{3/2}}\;$$

and


$$K_{WN}=-\frac{{\left(-\kappa^{2}\tau +\kappa \kappa_{s}+\tau \tau_{s}\right)}^{2}}{{\left({\left(\left(u-\kappa \right)\tau +\kappa_{s}\right)}^{2}+{\left(-u\kappa +\tau_{s}\right)}^{2}\right)}^{2}},\;\;H_{WN}=\frac{\kappa^{3}\tau^{2}+u{\kappa_{s}}^{2}-u\tau^{2}\tau_{s}+u{\tau_{s}}^{2}-\kappa^{2}\left(\tau \left(u\tau +\kappa_{s}\right)-u\tau_{s}\right)+\tau_{s}\kappa_{ss}-\kappa_{s}\tau_{ss}\;+\tau \left(\kappa_{s}\left(u^{2}-2\tau_{s}\right)-u\tau_{ss}\right)+\kappa \left(u^{2}\tau^{2}-\tau_{s}\left(u^{2}+\tau_{s}\right)-u\kappa_{ss}+\tau \left(u\kappa_{s}+\tau_{ss}\right)\right)\;}{2{\left({\left(\left(u-\kappa \right)\tau +\kappa_{s}\right)}^{2}+{\left(-u\kappa +\tau_{s}\right)}^{2}\right)}^{3/2}}.\;$$

From this, it follows that for κ is a nonzero constant and τ=0, we obtain $K_{NW}=K_{WN}=0$. On the other hand, since $H_{NW}\neq 0$, it can be concluded that the *NW* partner-ruled surfaces are not simultaneously minimal. □

**Theorem 15.** *Let* $P_{W}^{N}$ *and* $P_{N}^{W}$ *be NW partner-ruled surfaces, then the s–parameter curves of these surfaces are not simultaneously geodesic and asymptotic.*

*Proof*: The proof is carried out in a similar manner to the proof of the theorem given for the *TN* partner-ruled surfaces. □

**Theorem 16.** *Let* $P_{W}^{N}$ *and* $P_{N}^{W}$ *be NW partner-ruled surfaces, then the u–parameter curves of these surfaces are simultaneously geodesic and asymptotic.*

*Proof*: This proof employs the same methodology as the one used for the theorem concerning the *TN* partner-ruled surfaces. □

**Fig 1:**
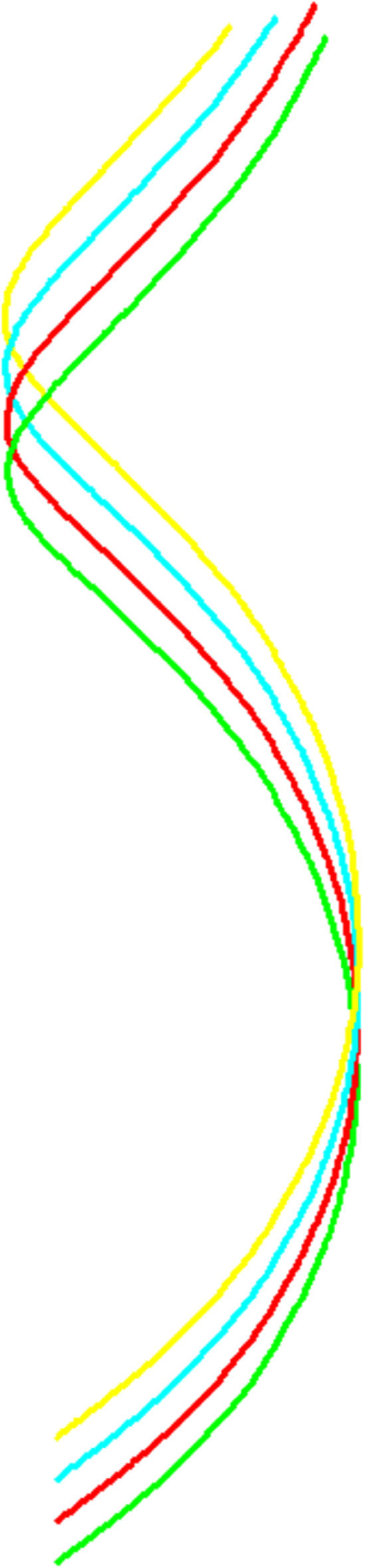
Evolution of α(s,t) evolved for s∈(−5,5) and t∈{1,2,3,4}.

**Example 1.** *Consider the evolving unit speed curve* $\alpha \left(s,t\right)=\left(3\cos\left(\frac{s}{5}\right),3\sin\left(\frac{s}{5}\right),\frac{4s}{5}+t\right)$ *with arc length parameter s and time parameter t, see* Fig 1.

*The Frenet frame elements and Darboux vector of* α(s,t) *are obtained as*


$$T\left(s,t\right)=\left(-\frac{3}{5}\sin\left(\frac{s}{5}\right),\frac{3}{5}\cos\left(\frac{s}{5}\right),\frac{4}{5}\right),N\left(s,t\right)=\left(-\cos\left(\frac{s}{5}\right),-\sin\left(\frac{s}{5}\right),0\right),\;B\left(s,t\right)=\left(\frac{4}{5}\sin\left(\frac{s}{5}\right),-\frac{4}{5}\cos\left(\frac{s}{5}\right),\frac{3}{5}\right),\;\;W\left(s,t\right)=\left(0,0,\frac{1}{5}\right)\;\kappa \left(s,t\right)=\frac{3}{25},\;\tau \left(s,t\right)=\frac{4}{25}.\;$$

**Fig 2:**
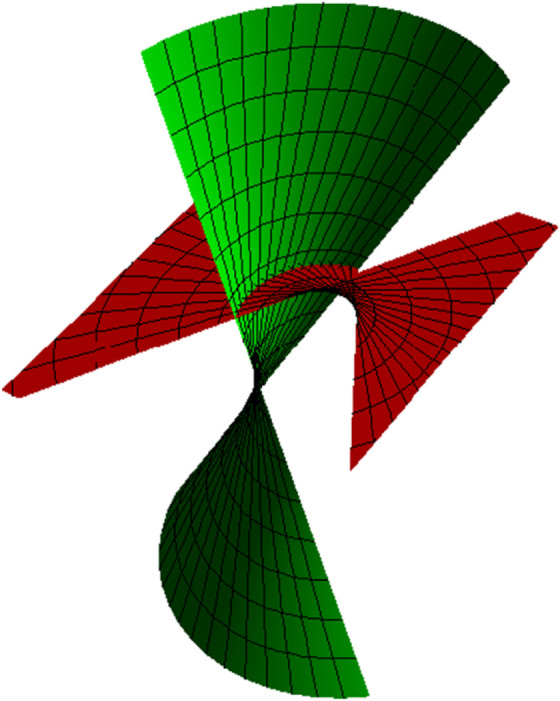
TN partner-ruled surfaces $P_{N}^{T}$ (red) and $P_{T}^{N}$ (green) for s∈(−5,5), u∈(−5,5) and *t* = 1.

**Fig 3:**
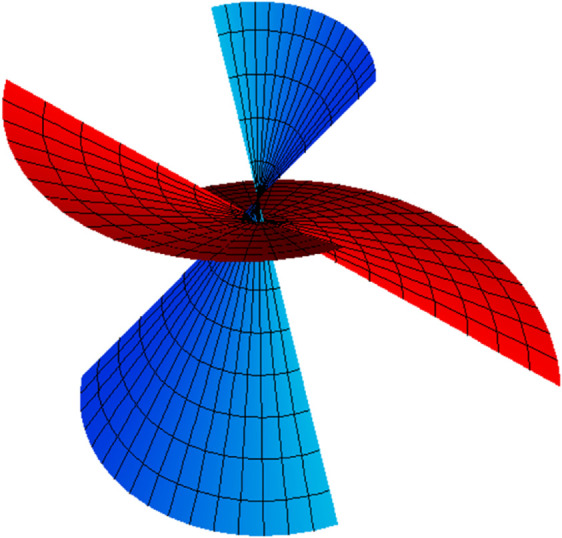
TB partner-ruled surfaces $P_{B}^{T}$ (red) and $P_{T}^{B}$ (cyan) for s∈(−5,5), u∈(−5,5) and *t* = 1.

**Fig 4:**
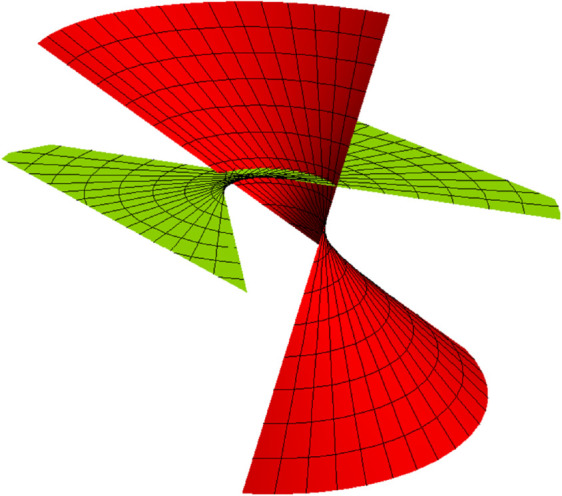
NB partner-ruled surface $P_{B}^{N}$ (red) and $P_{N}^{B}$ (yellow) for s∈(−5,5), u∈(−5,5) and *t* = 1.

**Fig 5:**
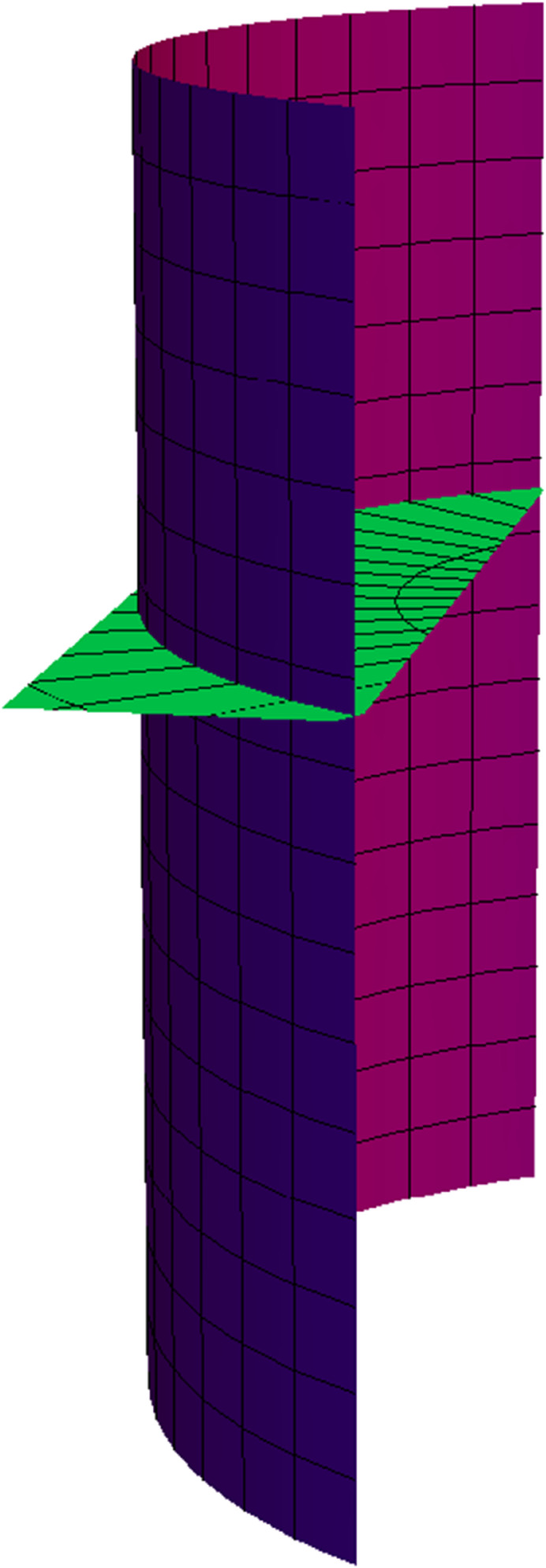
NW partner-ruled surface $P_{W}^{N}$ (purple) and $P_{N}^{W}$ (green) for s∈(−5,5), u∈(−5,5) and *t* = 1.

*The evolving generator curve* α(s,t) *allows forming the partner-ruled surfaces of the types of TN (*Fig 2), *TB (*Fig 3), *NB (*Fig 4), *and WN (Fig 5)*.

*Their parametric representations are as follows, respectively.*


$$\{P_{N}^{T}\left(s,u,t\right)=\left(-\frac{3}{5}\sin\left(\frac{s}{5}\right)-u\cos\left(\frac{s}{5}\right),\frac{3}{5}\cos\left(\frac{s}{5}\right)-u\sin\left(\frac{s}{5}\right),\frac{4}{5}\right),\;P_{T}^{N}\left(s,u,t\right)=\left(-\frac{3}{5}u\sin\left(\frac{s}{5}\right)-\cos\left(\frac{s}{5}\right),\frac{3}{5}u\cos\left(\frac{s}{5}\right)-\sin\left(\frac{s}{5}\right),\frac{4u}{5}\right),\;\;$$

$$\{P_{B}^{T}\left(s,u,t\right)=\frac{1}{5}\left(\left(-3+4u\right)\sin\left(\frac{s}{5}\right),\left(3-4u\right)\cos\left(\frac{s}{5}\right),\left(4+3u\right)\right),\;P_{T}^{B}\left(s,u,t\right)=\frac{1}{5}\left(\left(4-3u\right)\sin\left(\frac{s}{5}\right),\left(-4+3u\right)\cos\left(\frac{s}{5}\right),\left(3+4u\right)\right),\;\;$$

$$\{P_{B}^{N}\left(s,u,t\right)=\left(-\cos\left(\frac{s}{5}\right)+\frac{4}{5}u\sin\left(\frac{s}{5}\right),-\frac{4}{5}u\cos\left(\frac{s}{5}\right)-\sin\left(\frac{s}{5}\right),\frac{3u}{5}\right),\;P_{N}^{B}\left(s,u,t\right)=\left(-u\cos\left(\frac{s}{5}\right)+\frac{4}{5}\sin\left(\frac{s}{5}\right),-\frac{4}{5}\cos\left(\frac{s}{5}\right)-u\sin\left(\frac{s}{5}\right),\frac{3}{5}\right),\;\;\{P_{W}^{N}\left(s,u,t\right)=\left(-\cos\left(\frac{s}{5}\right),-\sin\left(\frac{s}{5}\right),\frac{u}{5}\right),\;P_{N}^{W}\left(s,u,t\right)=\left(-u\cos\left(\frac{s}{5}\right),-u\sin\left(\frac{s}{5}\right),\frac{1}{5}\right).\;\;$$

*Their first fundamental forms of these partner-ruled surfaces, respectively, have the coefficients*


$$\{E_{TN}=\frac{1}{625}\left(9+25u^{2}\right),\;\;F_{TN}=\frac{3}{25},\;G_{TN}=1,\;E_{NT}=\frac{1}{625}\left(25+9u^{2}\right),\;\;F_{NT}=-\frac{3}{25},\;G_{NT}=1,\;\;$$

$$\{E_{TB}=\frac{1}{625}{\left(3-4u\right)}^{2},\;\;F_{TB}=0,\;G_{TB}=1,\;E_{BT}=\frac{1}{625}{\left(4-3u\right)}^{2},\;\;F_{BT}=0,\;G_{BT}=1,\;\;$$

$$\{E_{NB}=\frac{1}{625}\left(25+16u^{2}\right),\;\;F_{NB}=\frac{4}{25},\;G_{NB}=1,\;E_{BN}=\frac{1}{625}\left(16+25u^{2}\right),\;\;F_{BN}=-\frac{4}{25},\;G_{BN}=1,\;\;$$

$$\{E_{NW}=\frac{1}{25},\;\;F_{NW}=0,\;G_{NW}=\frac{1}{25},\;E_{WN}=\frac{u^{2}}{25},\;\;F_{WN}=0,\;G_{WN}=1.\;\;$$

*Since they remain constant with respect to t, each of the partner-ruled surfaces is simultaneously inextensible.*

**Example 2.** *Consider the evolving unit speed curve*


$$\beta \left(s,t\right)=\frac{1}{\sqrt{2}}\left(\frac{\sin\left(s\left(1+t\right)\right)}{t+1},\frac{\cos\left(s\left(1+t\right)\right)}{t+1},s\right)$$

**Fig 6:**
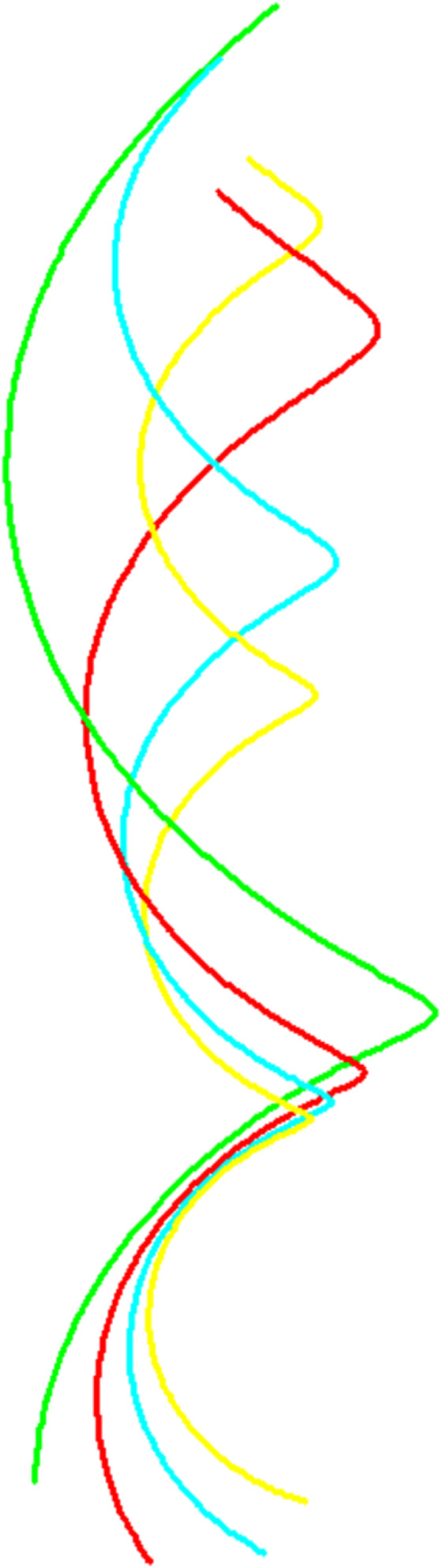
The evolving curve of β(s,t) evolved for s∈(−1,5) and t∈{1,2,3,4}.

*with arc length parameter s and time parameter t, see* Fig 6.

*The Frenet frame elements and Darboux vector of* β(s,t) *are obtained as*


$$T\left(s,t\right)=\left(\frac{\cos\left(s\left(1+t\right)\right)}{\sqrt{2}},-\frac{\sin\left(s\left(1+t\right)\right)}{\sqrt{2}},\frac{1}{\sqrt{2}}\right),\;N\left(s,t\right)=\left(-\sin\left(s\left(1+t\right)\right),-\cos\left(s\left(1+t\right)\right),0\right),\;B\left(s,t\right)=\left(\frac{\cos\left(s\left(1+t\right)\right)}{\sqrt{2}},-\frac{\sin\left(s\left(1+t\right)\right)}{\sqrt{2}},-\frac{1}{\sqrt{2}}\right),\;\;W\left(s,t\right)\;=\left(0,0,-1-t\right),\;\;\kappa \left(s,t\right)=\frac{1+t}{\sqrt{2}},\;\tau \left(s,t\right)=-\frac{1+t}{\sqrt{2}}.\;$$

**Fig 7:**
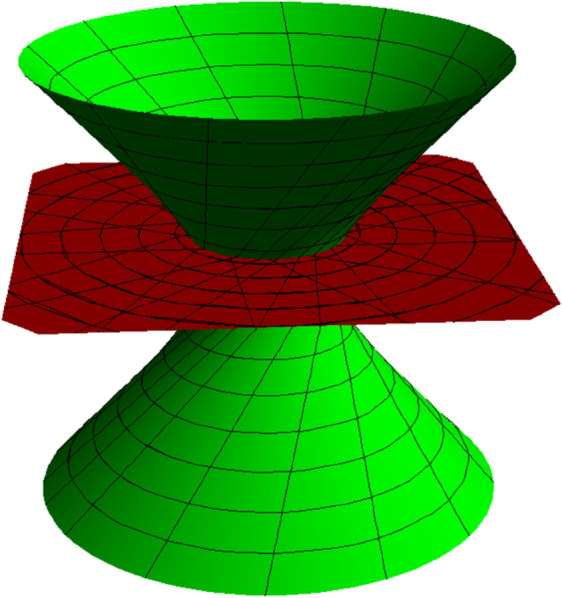
TN partner-ruled surfaces $P_{N}^{T}$ (red) and $P_{T}^{N}$ (green) for s∈(−1,5), u∈(−5,5), and *t* = 1.

**Fig 8:**
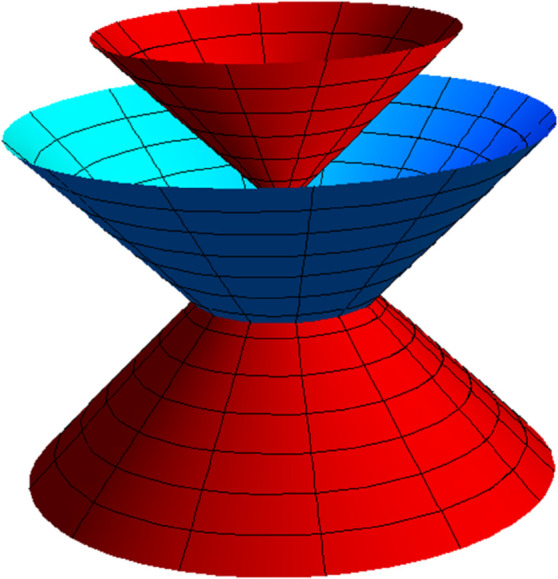
TB partner-ruled surfaces $P_{B}^{T}$ (red) and $P_{T}^{B}$ (cyan) for s∈(−1,5), u∈(−5,5), and *t* = 1.

**Fig 9:**
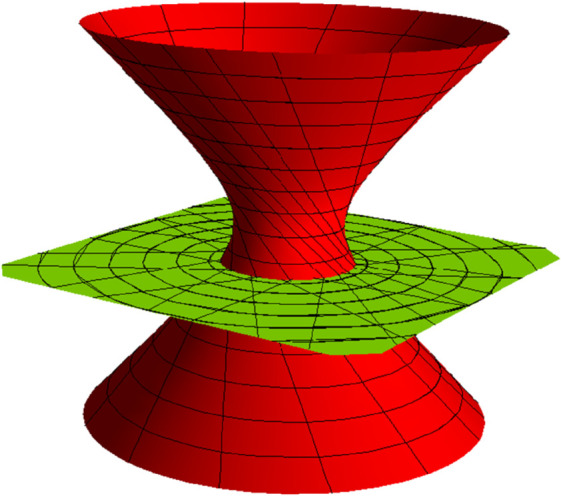
NB partner-ruled surface $P_{B}^{N}$ (red) and $P_{N}^{B}$ (yellow) for s∈(−1,5), u∈(−5,5), and *t* = 1.

**Fig 10:**
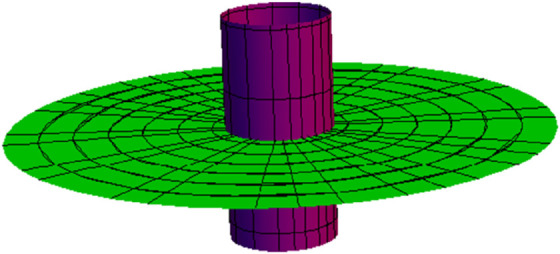
NW partner-ruled surface $P_{W}^{N}$ (purple) and $P_{N}^{W}$ (green) for s∈(−1,5), u∈(−5,5), and *t* = 1.


*The generator curve* β(s,t) *allows us to form the partner-ruled surfaces of the types of TN (*Fig 7), *TB (*Fig 8), *NB (*Fig 9), *and WN (Fig 10) with the parametric representations:*

$$\{P_{N}^{T}\left(s,u,t\right)=\frac{1}{\sqrt{2}}\left(\cos\left(s\left(1+t\right)\right)-\sqrt{2}u\sin\left(s\left(1+t\right)\right),-\sin\left(s\left(1+t\right)\right)-\sqrt{2}u\cos\left(s\left(1+t\right)\right),1\right),\;P_{T}^{N}\left(s,u,t\right)=\frac{-1}{\sqrt{2}}\left(\sqrt{2}\sin\left(s\left(1+t\right)\right)-u\cos\left(s\left(1+t\right)\right),\sqrt{2}\cos\left(s\left(1+t\right)\right)+u\sin\left(s\left(1+t\right)\right),-u\right).\;\;$$

$$\{P_{B}^{T}\left(s,u,t\right)=\frac{1}{\sqrt{2}}\left(\left(1+u\right)\cos\left(s\left(1+t\right)\right),-\left(1+u\right)\sin\left(s\left(1+t\right)\right),1-u\right),\;P_{T}^{B}\left(s,u,t\right)=\frac{1}{\sqrt{2}}\left(\left(1+u\right)\cos\left(s\left(1+t\right)\right),-\left(1+u\right)\sin\left(s\left(1+t\right)\right),-1+u\right),\;\;$$

$$\{P_{B}^{N}\left(s,t,u\right)=\frac{-1}{\sqrt{2}}\left(\sqrt{2}\sin\left(s\left(1+t\right)\right)-u\cos\left(s\left(1+t\right)\right),\sqrt{2}\cos\left(s\left(1+t\right)\right)+u\sin\left(s\left(1+t\right)\right),u\right),\;P_{N}^{B}\left(s,t,u\right)=\frac{-1}{\sqrt{2}}\left(-\cos\left(s\left(1+t\right)\right)+\sqrt{2}u\sin\left(s\left(1+t\right)\right),\sin\left(s\left(1+t\right)\right)+\sqrt{2}u\cos\left(s\left(1+t\right)\right),1\right),\;\;$$

*and*


$$\{P_{W}^{N}\left(s,t,u\right)=\left(-\sin\left(s\left(1+t\right)\right),-\cos\left(s\left(1+t\right)\right),-\left(1+t\right)u\right),\;P_{N}^{W}\left(s,t,u\right)=\left(-u\sin\left(s\left(1+t\right)\right),-u\cos\left(s\left(1+t\right)\right),-1-t\right).\;\;$$

*Their first fundamental forms of these partner-ruled surfaces, respectively, have the coefficients*


$$\{E_{TN}=\frac{1}{2}{\left(1+t\right)}^{2}\left(1+2u^{2}\right),\;\;F_{TN}=\frac{1+t}{\sqrt{2}},\;G_{TN}=1,\;E_{NT}=\frac{1}{2}{\left(1+t\right)}^{2}\left(2+u^{2}\right),\;\;F_{NT}=-\frac{1+t}{\sqrt{2}},\;G_{NT}=1,\;\;$$

$$\{E_{TB}=\frac{1}{2}{\left(1+t\right)}^{2}{\left(1+u\right)}^{2},\;\;F_{TB}=0,\;G_{TB}=1,\;E_{BT}=\frac{1}{2}{\left(1+t\right)}^{2}{\left(1+u\right)}^{2},\;\;F_{BT}=0,\;G_{BT}=1,\;\;$$

$$\{E_{NB}=\frac{1}{2}{\left(1+t\right)}^{2}\left(2+u^{2}\right),\;\;F_{NB}=-\frac{1+t}{\sqrt{2}},\;G_{NB}=1,\;E_{BN}=\frac{1}{2}{\left(1+t\right)}^{2}\left(1+2u^{2}\right),\;\;F_{BN}=\frac{1+t}{\sqrt{2}},\;G_{BN}=1,\;\;$$

*and*


$$\{E_{NW}={\left(1+t\right)}^{2},\;\;F_{NW}=0,\;G_{NW}={\left(1+t\right)}^{2},\;E_{WN}={\left(1+t\right)}^{2}u^{2},\;\;F_{WN}=0,\;G_{WN}=1.\;\;$$

*We have differentiated the last equations with respect to time t, we have*


$$\{{\left(E_{TN}\right)}_{t}\neq 0,\;\;\;{\left(F_{TN}\right)}_{t}\neq 0,\;\;{\left(G_{TN}\right)}_{t}=0,\;{\left(E_{NT}\right)}_{t}\neq 0,\;\;\;{\left(F_{NT}\right)}_{t}\neq 0,\;\;{\left(G_{NT}\right)}_{t}=0,\;\;$$

$$\{{\left(E_{TB}\right)}_{t}\neq 0,\;\;{\left(F_{TB}\right)}_{t}=0,{\left(G_{TB}\right)}_{t}=0,\;{\left(E_{BT}\right)}_{t}\neq 0,\;\;{\left(F_{BT}\right)}_{t}=0,\;{\left(G_{BT}\right)}_{t}=0,\;\;$$

$$\{{\left(E_{NB}\right)}_{t}\neq 0,\;\;\;{\left(F_{NB}\right)}_{t}\neq 0,\;\;{\left(G_{NB}\right)}_{t}=0,\;{\left(E_{BN}\right)}_{t}\neq 0,\;\;\;{\left(F_{BN}\right)}_{t}\neq 0,\;\;{\left(G_{BN}\right)}_{t}=0,\;\;$$

*and*


$$\{{\left(E_{NW}\right)}_{t}\neq 0,\;\;\;{\left(F_{NW}\right)}_{t}\neq 0,\;\;{\left(G_{NW}\right)}_{t}=0,\;{\left(E_{WN}\right)}_{t}\neq 0,\;\;\;{\left(F_{WN}\right)}_{t}\neq 0,\;\;{\left(G_{WN}\right)}_{t}=0.\;$$

*These mean that none of the partner-ruled surfaces generated by vectors of Frenet and Darboux are simultaneously inextensible.*

## Conclusions

This study explores the simultaneous inextensible evolutions of partner-ruled surfaces generated by a pair of vectors from the set of the tangent, principal normal, binormal, and Darboux vectors of an evolving space curve. By these explored structures, the articulated can be designed for various complex tasks as follows: a pair of rigid links of the arm sweeps out a pair of ruled surfaces with a certain rule as they rotate or translate simultaneously. So the motions of a pair of links can be modeled as the evolutions of a partner-ruled surface. If the pair of robotic links cannot stretch, bend, or warp, this also requires inextensibility. Thus, the obtained conditions for the evolving partner-ruled surfaces to be inextensible, developable, or minimal may be useful for such robotic arms.
